# Effect of Laser Processing Parameters on Microstructure, Hardness and Tribology of NiCrCoFeCBSi/WC Coatings

**DOI:** 10.3390/ma14206034

**Published:** 2021-10-13

**Authors:** Jelena Škamat, Olegas Černašėjus, Gulnara Zhetessova, Tatyana Nikonova, Olga Zharkevich, Nikolaj Višniakov

**Affiliations:** 1 Laboratory of Composite Materials, Faculty of Civil Engineering, Vilnius Gediminas Technical University, 08217 Vilnius, Lithuania; 2 Department of Mechanics and Materials Engineering, Faculty of Mechanics, Vilnius Gediminas Technical University, 03224 Vilnius, Lithuania; olegas.cernasejus@vilniustech.lt (O.Č.); nikolaj.visniakov@vilniustech.lt (N.V.); 3 Department of Technological Equipment, Engineering and Standardization, Faculty of Mechanical Engineering, Karaganda Technical University, Karaganda 100000, Kazakhstan; zhetesova@mail.ru (G.Z.); nitka82@mail.ru (T.N.); zharkevich82@mail.ru (O.Z.)

**Keywords:** NiCrCoFeCBSi/WC coatings, laser processing, thermal spray, microstructure, hardness, tribology

## Abstract

In the present study, pulsed laser post-processing was applied to improve the properties of the thermally sprayed NiCrCoFeCBSi/40 wt.% WC coatings. The powder mix was deposited onto a mild steel substrate by flame spray method and then the as-sprayed coatings were processed by Nd:YAG laser. The peak power density applied was between 4.00 × 10^6^ and 5.71 × 10^6^ W/cm^2^, and the laser operating speed ranged between 100 and 400 mm/min, providing processing in a melting mode. Scanning electron microscopy, energy dispersive spectroscopy, Knop hardness measurements, and “ball-on-disc” dry friction tests were applied to study the effect of the processing parameters on the geometry of laser pass and microstructure, hardness, and tribology of the processed layers. The results obtained revealed that pulsed laser processing provides a monolithic remelted coating layer with the microstructure of ultrafine, W-rich dendrites in Ni-based matrix, where size and distribution of W-rich dendrites periodically vary across remelted layer depth. The composition of W-rich dendrites can be attributed to a carbide of type (W, Cr, Ni, Fe)C. The cracks sensitivity of coatings was visibly reduced with the reduction of power density applied. The hardness of coatings was between ~1070 and ~1140 HK0.2 and correlated with microstructure size, being dependent on the processing parameters. The friction coefficient and wear rate of coatings during dry sliding were reduced by up to ~30% and up to ~2.4 times, respectively, after laser processing.

## 1. Introduction

Ni alloy–WC metal matrix composite (MMC) coatings are widely used in various fields of industry and technology to protect the working surfaces of parts from corrosion and wear, which is primarily due to the unique properties of nickel itself and the excellent properties of tungsten carbide as a hardening phase, as well as their good compatibility—WC is well wetted by nickel alloy melts [1]. Ni alloys possess high strength and toughness along with a resistance to corrosion and wear in a wide range, which is mainly predetermined by the alloying system [2]. WC possesses a high melting point (2600–2850 °C), high fracture toughness (28 MPa∙m^1/2^), and high hardness (16–22 GPa) [3]. It is widely recognized, as well, that cemented tungsten carbide, which is usually used in coatings, has some degree of plasticity, especially when compressive loads are applied [4]. Moreover, it is shown that at the later deformation stage dislocations’ density of WC in cermet dominates, indicating a more important WC role in the plasticity than a role of a metal binder [5]. This gives an advantage to WC over brittle hard materials.

For depositing protective Ni–WC coatings, thermal spraying methods are widely used, including flame spraying (FS), plasma transferred arc (PTA) spray, arc spraying, and high-velocity oxygen fuel (HVOF) spray—mainly using powder materials. In the last two decades, the main attention has been paid to the development of laser methods for the deposition of Ni–WC coatings [6,7,8,9,10,11,12,13,14,15,16,17,18,19,20,21,22,23,24,25,26,27,28,29,30]. Two main methods can be distinguished—laser remelting of previously sprayed layers [6,7,8] and, actually, coating formation using laser beam energy (also known as laser cladding) [9,10,11,12,13,14,15,16,17,18,19,20,21,22,23,24,25,26,27,28,29,30].

During the laser cladding, the stream of powdered material or wire is fed onto the substrate surface (or powder is preplaced there) under the laser beam, which generates a melted pool. The laser moves over the surface and forms a narrow coating layer by one individual pass. To coat the entire surface, the process typically is performed pass-by-pass with some pass overlapping. As is pointed out in almost all the publications, laser cladding, compared with conventional spraying methods (FS, PTA, HVOF, etc.), enables obtaining denser, low-porous, strongly bonded to the substrate layers with minimal dilution, heat-affected zone and substrate distortion. Moreover, it is known that due to extremely high heating/cooling regimes, laser processing produces different microstructures and properties that cannot be obtained utilizing conventional deposition techniques. However, some disadvantages are typical for laser cladding, including dissolution of WC particles in molten Ni-based matrix, resulting in hardness drop and increased coating porosity; sinking of high-density WC particles to the coating bottom forming, respectively, a nonhomogenous layer with the weakened top layer of the coating; crack formation due to thermal stress. The publications available in the field present various ways to solve mentioned problems, such as parameters optimization [18,22,23,25], substrate preheating [16,17,18,21,22,27], formation of gradient coatings [22], addition of rare-earth elements and other compounds [10,12,14,16,21,30], and vibration [12,29].

Tungsten carbide is used in composite coatings mainly to increase the hardness and provide high wear resistance, which cannot be obtained using metal alloy coats. However, not all techniques showed in the publications allowed reaching such results. In the works [16,17,21,22,27], where authors used substrate preheating to a temperature from ~200 °C and up, the following best hardness results were reported: 760 HV at 59 wt.% WC in powder mixture [16], 1350 HV0.2 (for WC phase) [21], 740 HV0.2 at 10 wt.% WC [22], 1200 HV0.2 at 50 wt.% WC [27]. Zhou et al., in their work [18], obtained uniform ~1100 HV0.2 hardness using laser induction hybrid rapid cladding (LIHRC) method with substrate preheating to a temperature of ~1173 K. In the other works, where authors studied the effect of vibration [12,29] and addition of rare-earth element-containing oxides such as La_2_O_3_ and CeO_2_ [10,12,14], the following hardness values are given: ~500 HV0.2 at 60 wt.% WC [10]; 55 HRC/or 650 HV at 60 wt.% WC [12]; ~525 HV0.2 at ~50 wt.% WC [14]; ~1100 HV0.2 at 10 wt.% WC [29]. Recently, Zhao et al. [30] proposed a novel hot-wire method for the deposition of Ni/WC composite coatings. As the results presented show, dissolution of WC particles in Ni-based matrix was successfully inhibited and homogenous layers were obtained; however, hardness of deposited coatings was not so high—slightly above 600 HV0.2 at 45 wt.% WC.

Summarizing the results presented in the abovementioned works, it can be argued that some progress has been achieved in the development of laser cladding technologies, and some solutions can already be applied in the industry. However, in some reported cases, the hardness of obtained coatings (despite using high WC content) was not so high. Some other nuances can be noted as well: using the laser cladding method with powder preplaced on the substrate surface, it is difficult to implement the process and to provide even thickness of preplaced powder layer for parts of more complex geometry with surfaces lying in different planes. Applying the method with automatic powder feeding, the result also depends on the method and place of powder feeding, which somewhat complicates the procedure for optimizing the parameters for each specific case. In addition, it should be noted that many of the studies presented use nonstandard laboratory equipment, the availability of which is limited. From this point of view, the remelting of the already formed coating, when a coating is firstly being sprayed onto the substrate by standard spraying technique and then remelted by laser, is simpler—standard laser installations can be used, for example, welding, the cost of which has a significantly decreased recently, which makes them available to a wider range of consumers. The works [6,7,8] present the results of using a CO_2_ laser remelting technique to postprocess HVOF and plasma-sprayed deposits. Authors pointed out improvement in corrosion [6,7,8], wear [7], and tribological [8] performance of the obtained layers, related to favorable changes in phase composition and microstructure along with decreased porosity. The hardness of HVOF coatings reached 1277 HV using powder containing ~70 wt.% WC [7], while for plasma-sprayed layers, only 280 HV0.2 hardness at ~88 wt.% WC is reported [6]. It should be noted, as well, that HVOF is a comparably expensive deposition method and in combination with laser processing, it is becoming expensive. In the present work, a less expensive and simple to operate flame spray method is utilized for primary deposition of the NiCrCoFeCBSi–WC composite coating followed by remelting using pulsed Nd:YAG laser. The flame-spray method is especially easy to automate for cylindrical parts; manually or using a standard robot-manipulator, it is possible to deposit a coating on the surfaces of more complex geometry. Using the hand of a manipulator for the laser head movement, the remelting process may be performed for a surface of various complexity. Flame-sprayed deposits provide stable coating thickness and uniform distribution of WC particles across the coating [31]. They are normally more porous and less adhered to a substrate compared with HVOF or PTA; however, subsequent laser remelting eliminates porosity and provides metallurgical bonds.

In our previous article [31], we presented the results of comparing flame-sprayed Ni-based/WC coatings obtained by remelting by conventional methods (flame, furnace, induction) and a laser beam using Nd:YAG laser with wavelength 1064 nm. The microstructure and hardness of the conventionally remelted coatings were similar and were mainly predetermined by the heating duration. The optimal microstructure, providing the highest hardness ~880 HK2.0 and the best wear resistance, was obtained in a narrow heating duration interval when the metal matrix was fused enough to wet substrate surface and fill pores and gaps between sprayed splats, while WC particles retained their initial state and were not dissolved in a metal matrix. The conventionally remelted coatings were directly compared with a laser-remelted one, which was processed in a regime, providing the coating melting over the entire coating thickness and with some melting of the substrate to form sufficient bond between the coating and substrate. It was found that pulsed laser processing formed ultrafine W-rich dendrites in Ni-rich matrix microstructure, which differed from that of conventionally remelted coatings by morphology, size, and phase composition. This resulted in a visible improvement of hardness (~12–31%) and wear resistance (up to 42% by the thickness of the removed layer), as compared with the best results of flame, induction, and furnace heating. However, a wider study of the layers, laser-remelted at various parameters, was not carried out. At the same time, the results of the abovementioned works showed that the microstructure and properties of the coatings may vary significantly, both using different laser types and varying processing parameters, which is related, first of all, with the difference in a heating/cooling regime. Thus, it was shown in [7] that the hardness of a remelted HVOF coating increased and its wear rate and friction coefficient decreased with the decrease in a laser scanning rate, which is associated with a higher level of the coating’s densification and reduced porosity at higher heat input. Li et al. demonstrated, in their study [32], the increase in coating’s hardness with a decrease of applied laser power, attributed to a formation of a higher fraction of amorphous phase and finer microstructure. These results show, as well, that two different laser processing regimes may be considered regarding obtained dependencies between processing parameters and coatings’ properties: less powered heating, resulting in coating’s densification due to partial melting of phases having a low melting point, and more powered heating, providing temperatures signally above melting point with followed crystallization from the fully melted state. The trends of dependencies may differ for these two regimes. Since, to date, there is a lack of results on the millisecond pulsed laser remelting of the flame-sprayed Ni-based/WC coatings, in the present work, a more detailed report on the influence of pulsed Nd:YAG laser processing parameters on the pass geometry, microstructure, and properties of the processed layers is presented.

## 2. Materials and Methods

**Figure 1:**
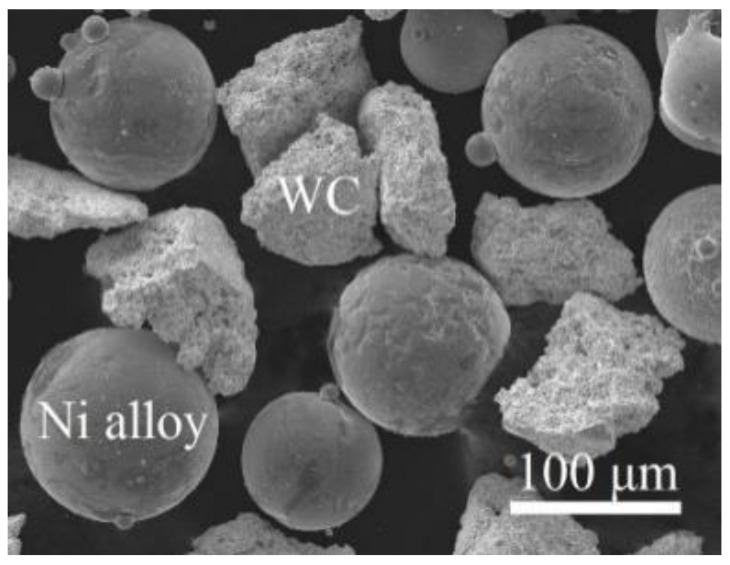
SEM micrograph of the powder used for coatings spray.

The mixture of self-fluxing Ni-based alloy powder (chemical composition in wt.% declared by manufacturer: 0.4 C; 13.8 Cr; 3.9 Fe; 11.8 Co; 7.9 B + Si; Ni—balance) and 40 wt.% WC powder was used for the formation of the coatings; the particles’ size was between 38 and 125 μm. Figure 1 shows the general view of the powder used. Structural steel S235 plates of dimensions 150 mm × 40 mm × 8 mm were used as a substrate. Since the effect of a particular substrate’s material on the coatings’ composition and properties was not studied in this paper, cheap, widely available, low-carbon steel grade, which is used as a structural material for various purposes, was chosen. The primary deposition of the coatings was performed employing the oxy-fuel flame spray technique. Rototec 80 (Castolin Eutectic, Lausanne, Switzerland) spraying equipment was used. The main parameters of the deposition process were as follows: spraying distance ~170 mm; torch operating speed 250 mm/s; a space between adjacent passes 5 mm; neutral oxygen–acetylene flame; the substrate was flame preheated to a temperature of ~230–250 °C; the final thickness of the coating was obtained by eight sprayed layers and was ~1.2–1.3 mm (the maximal recommended thickness is 1.5 mm). To provide stable process parameters and uniform deposits, a manipulator Motoman 100 (Yaskawa Nordic, Torsås, Sweden) was employed. Before spraying, the substrate surface was cleaned with isopropyl alcohol, grit-blasted, again washed with isopropyl alcohol, and dried in hot air. The sprayed coatings were then laser-processed using pulsed Nd:YAG laser (1064 nm wavelength; 6 ms pulse duration; 20 Hz frequency). The surface remelting was performed pass-by-pass, applying linear laser trajectory with 0.9 mm step between adjacent passes. The laser spot size was 0.3 mm, laser current varied between 120 and 170 A, and processing speed—between 100 and 400 mm/min, providing processing in a melting mode. More detailed information regarding the methodology for laser processing parameters calculation and used physical properties of the materials can be found in [31]. The summary for samples coding is presented in Table 1. As a reference, the as-sprayed coating was used, marked as R0.

The microstructural analysis was performed by scanning electron microscopy (SEM) employing a SEM JEOL JSM-7600F (JEOL, Tokyo, Japan) microscope equipped with an energy dispersive spectrometer IncaEnergy 350 (Oxford Instruments, Abingdon, UK) for X-ray microanalysis. The main analysis parameters: 10 kV accelerating voltage; ~8 mm working distance; mixed secondary and backscattered electrons signal for imaging. For the microscopic analysis, the samples were sectioned, mounted, ground, and polished by conventional methodic for metallographic analysis (last polishing step performed using 0.2 μm fumed silica suspension). The geometrical parameters (width and depth) of the melt pool of individual laser-processed passes were determined using SEM images along with micrographs obtained with optical microscope Nikon MA200 (Nikon, Tokyo, Japan) on the samples’ cross-sections. The averages of eight measurements are presented in the paper, along with standard deviation based on the entire population given as arguments, calculated using Microsoft Excel 2016 STDEV.P function.

Zwick Roell ZHμ microhardness tester (ZwickRoell GmbH & Co. KG, Ulm, Germany) was used for hardness study. Measurements were carried out by the Knoop method with 200 gr load and 10 s duration. The measurements of microhardness of coatings were carried out on prepolished surfaces of samples’ cross-sections. The average values of microhardness were calculated as an arithmetic mean of 12 measurements and are presented in the paper with standard deviation based on the entire population given as arguments, calculated using Microsoft Excel STDEV.P function. The paper presents the curves of the microhardness distribution across the thickness of the coatings (microhardness depth profiles) as well, which were obtained by indentation with a step of 50 μm, making indentations along the center line of melt pool cross-section of an individual pass.

The tribology study was performed on the processed surfaces by the “ball-on-disc” dry friction test using a Microtest tribometer (Microtest, S. A., Madrid, Spain). In the tribology study, the following test conditions were used: sliding distance *l*—100 m; sliding speed—200 rpm; radius of the trajectory—2 mm; load—40 N; temperature of the experiment—21 ± 1 °C; indentor—6 mm diameter ball made of tempered stainless steel AISI52100. Before the test, the surface of the samples was prepolished to *Ra* ~0.27 µm (the measurement across the grinding direction) and to *Ra* ~0.06 µm (the measurement along the grinding direction). The surface microroughness parameters of the tested samples were measured using a profilometer TR-200 with an accuracy ± 0.01 μm. “Precisa XR 205SMDR” (Precisa Gravimetrics AG, Dietikon, Switzerland) analytical balance with an accuracy of 0.00001 g was used to measure the mass of the samples before and after tribology test and to evaluate samples’ mass loss *D_m_*. The wear rate *m_w_* of the coatings was calculated by dividing the mass loss *D_m_* by sliding distance *l* and expressed in g/m. The average *m_w_* values of three tests are presented in the paper. The friction coefficients were calculated after the test data for the first 10 m of sliding distance was eliminated; the average values are presented.

## 3. Results

### 3.1. Effect of Processing Parameters on Pass Geometry and Cracks

#### 3.1.1. Effect of Laser Current and Operating Speed on Pass Geometry

**Figure 2:**
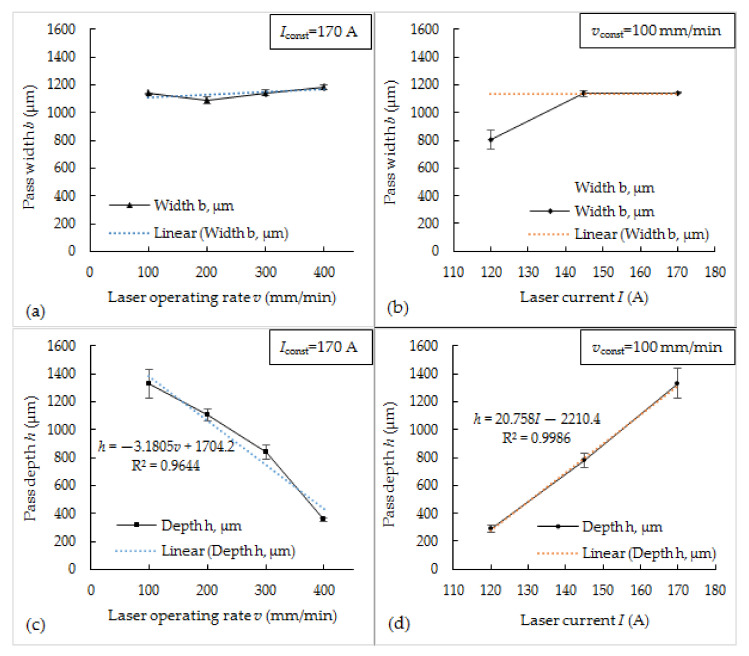
Pass width (**a**,**b**) and depth (**c**,**d**) dependencies on the laser operating rate (at a constant 170 A laser current) and laser current (at a constant operating rate 100 mm/min).

Figure 2 shows the dependencies of depth and width of laser-processed passes on the laser current and operating speed. It was found that the width of passes does not vary significantly with varying processing speed and ranges between ~1050 and ~1150 μm. When laser current was 145 A and 170 A, the pass width did not change as well; and only the reduction of laser current to 120 A led to a reduction of pass width by ~30%—from ~1140 μm to ~800 μm (Figure 2a,b). At the same time, pass depth ranged between ~290 and ~1330 μm and was in strong near-linear dependency on the processing parameters—directly proportional for a laser current and inversely proportional for laser operating speed (Figure 2c,d). When operating speed was increased by 4 times (from 100 to 400 mm/min), the pass depth was proportionally reduced by ~3.7 times, while an increase in laser current by ~40% led to an increase in pass depth by ~4.5 times.

The thickness of the laser-processed coatings was reduced, compared with as-sprayed one (~1.2–1.3 mm). The compaction of the deposit after remelting step is typical for spray-fuse technology. Here, the reduction of coating thickness with the increase in a melt pool depth was observed: the deeper the melt pool, the larger the part of the as-sprayed layer that was remelted and, respectively, the thinner the coating became. The thickness of processed coatings was as follows: for S1 samples—between ~1.03 and ~1.12 mm; S2—0.94–0.96 mm; S3—0.86–0.91 mm; S4—0.85–0.88 mm; S5—0.97–0.99 mm; S6—~1.02–1.10 mm.

#### 3.1.2. Effect of Power Density and Processing Speed on Coatings’ Cracking

**Figure 3:**
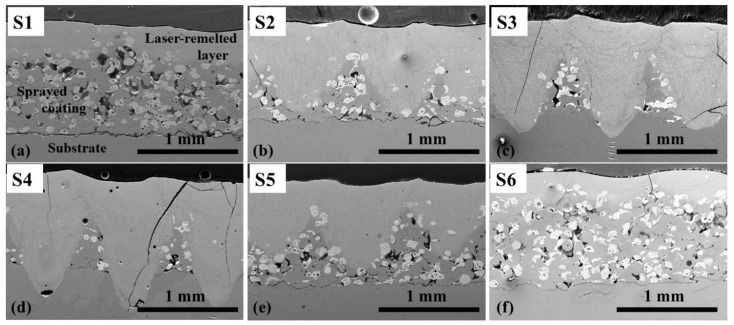
SEM micrographs of the coatings laser-processed at different parameters: (**a**) S1—170 A/400 mm/min; (**b**) S2—170 A/300 mm/min; (**c**) S3—170 A/200 mm/min; (**d**) S4—170 A/100 mm/min; (**e**) S5—145 A/100 mm/min; (**f**) S6—120 A/100 mm/min; deep transverse cracks over the entire layer of S4 and S3 coatings processed at 170 A laser current; visibly less cracks in coatings S1, S2, S5, and S6 processed applying lower heat input or lower power density.

As noted earlier, cracking of Ni–WC coatings during laser processing is one of the typical problems caused by thermal stresses. Cracks were also recorded on all the coatings obtained in this study (Figure 3). Sample S4, obtained using the highest laser current 170 A, determining the highest peak power density (5.71 × 10^6^ W/cm^2^), and the highest heat input, determined by the lowest operating speed (100 mm/min), showed the most intense cracking—deep transverse cracks were observed over the entire processed layer (Figure 3d). With decreasing current, a significant decrease in cracks was observed (Figure 3e,f), which is consistent with the conclusions presented by other authors [18]. The samples remelted at a lower heat input showed some decrease in cracks appearance as well (Figure 3a,b). It is possible that the application of the pre- and postheating procedures could reduce cracking of the coating even more.

### 3.2. Microstructural Analysis

#### 3.2.1. Brief Characterization of As-Sprayed Coating

**Figure 4:**
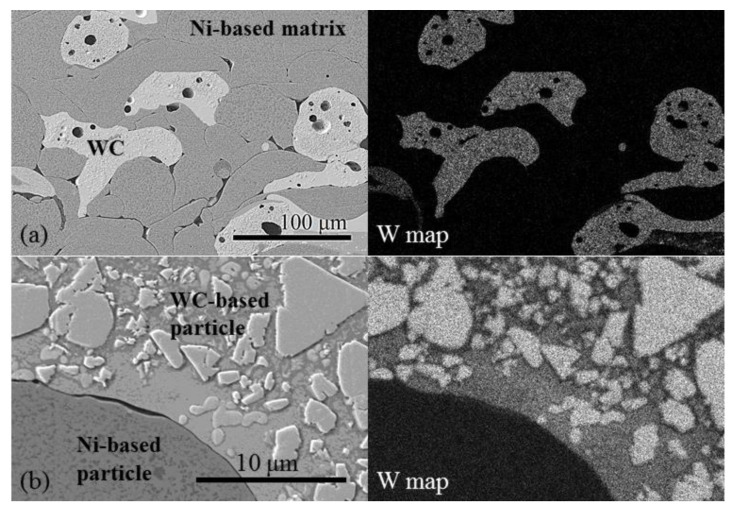
SEM micrographs of the as-sprayed coating (R0) at different magnifications, and tungsten (W) distribution maps obtained with EDS: (**a**) coating general view at a lower magnification and map of W distribution; (**b**) morphology of the WC-based particle at a higher magnification and map of W distribution; lighter areas correspond to a higher element concentration.

Figure 4a shows the microstructure of the as-sprayed Ni–WC coating: the Ni-based and WC-based splats, deformed in a lower or higher degree. In SEM micrograph (Figure 4a), WC-based particles are clearly identifiable in Ni-based matrix and confirmed as well by tungsten distribution map, obtained with EDS. At higher magnification, the morphology of WC-based particles can be observed (Figure 4b). According to EDS results, the trigonal, polygonal, and irregularly shaped W-rich particles of different sizes distributed in a Co-based matrix consist of tungsten and carbon in various ratios and at the presence of low content of oxygen (up to 1.4 wt.%) and cobalt (up to 0.6 wt.%).

#### 3.2.2. Microstructure of Laser-Processed Coatings

**Figure 5:**
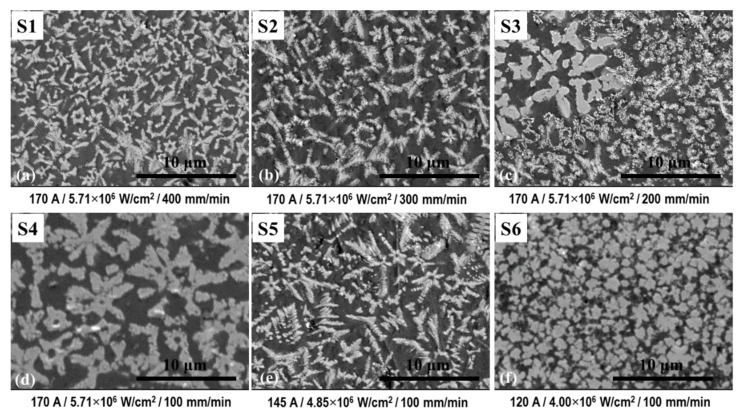
SEM micrographs of the laser-processed coatings S1–S6: the microstructure prevailing in the top layer of the coating; lighter inclusions—W-rich phases, darker matrix—γNi-based phase.

The microstructure of laser-processed layers differs strongly from that of the as-sprayed. As shown earlier [31], the cross-section of each pass exhibits a layered microstructure consisting of W-rich dendrites uniformly distributed in the Ni-rich matrix. When a pulsed laser is used, the melting process runs point-by-point with points overlapping, determined by the operating speed. As a result, the layered structure is formed with dendrites size varied within each layer. At the lower magnification, all the structures obtained after laser processing seem to be very similar. However, microscopic analysis at higher magnification showed the prevailing of different structures in top layers of coatings laser-processed at different parameters (Figure 5). It was found that at 170 A current and 400 mm/min processing speed, the size of W-rich dendrites was the smallest, as compared with other regimes (Figure 5a). With the reduction of speed to 300 mm/min, the slight increase in dendrites length can be observed, which can be associated with the increased heat input and changed heating–cooling regimes (Figure 5b). Further decrease in speed to 200 mm/min and 100 mm/min led to a formation of more thick and coarse dendrites (Figure 5c,d). The change of power density at constant operating speed (100 mm/min) had a certain effect on the prevailing microstructure as well. Thus, with the reduction of laser current from 170 A to 145 A, the thinning of the dendrites can be observed as well as a slight decrease in their length. At 120 A current, fine and near-equiaxed W-rich dendrites were found in the coating top layer.

**Figure 6:**
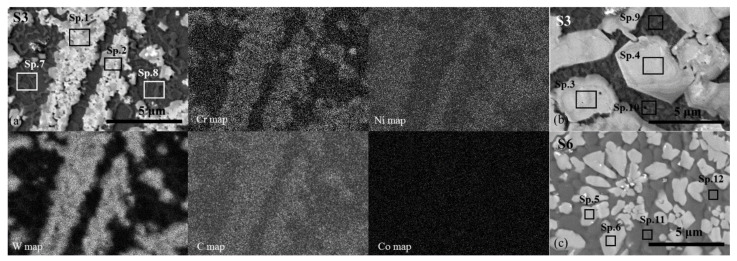
SEM micrographs of the laser-processed coatings S3 (**a**,**b**) and S6 (**c**) with denoted points of EDS analysis (Sp.1–Sp.12; see Table 2) and maps of the distribution of the elements in the area showed in (**a**).

The chemical microanalysis by EDS (point—in Table 2; maps—in Figure 6) showed that W-rich precipitates contain a significant amount of C, Cr, and Ni and a particular concentration of Fe. Besides prevailing microstructure, shown in Figure 5, regions containing W-rich precipitates of different sizes and shapes were observed in the top layer for all the samples as well. Figure 6 shows typical examples.

EDS revealed that the W-rich precipitates of different sizes and shapes do not differ visibly in their composition (Table 2, Sp.1–Sp.6). According to the atomic C ratio to metals, the W-rich phase can be identified as a complex carbide of MeC type, where Me corresponds to W + Cr + Ni + Fe. EDS analysis of Ni-based matrix showed that in coating areas, where fine dendritic tungsten carbides are formed (typically, applying 170 A laser current), the concentration of Co is slightly larger as compared with that of coatings areas with coarser tungsten carbides, possibly remaining due to noncomplete dissolution of primary WC-based particles (Table 2, Sp.7–Sp.12). Such increase, most likely, can be attributed to a more complete dissolution of WC particles and the mixing of binder components with Ni-based matrix at higher laser power. For S3 and S4 series, the increase in Fe concentration in matrix was observed as well, related to substrate melting and admixing.

### 3.3. Hardness of Laser-Processed Coatings

**Figure 7:**
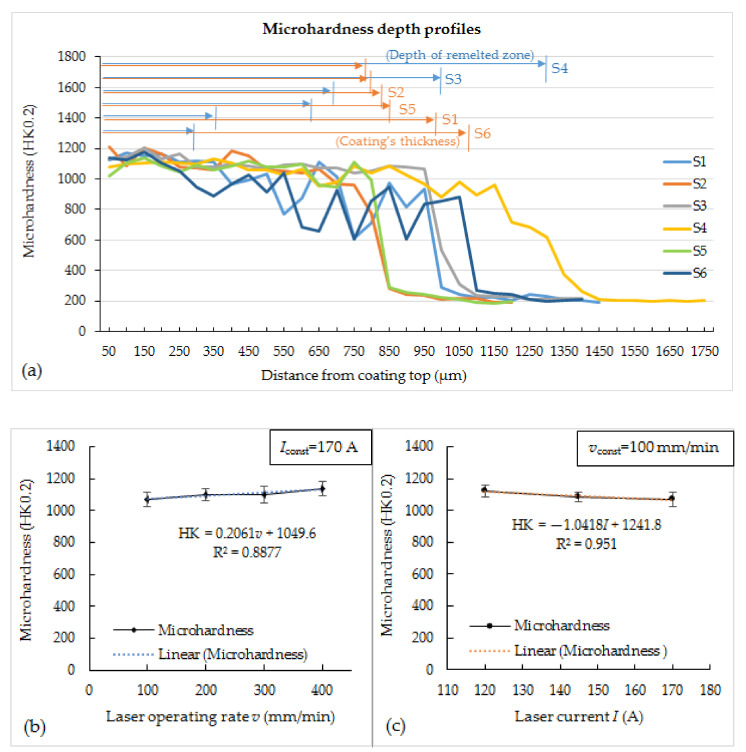
Microhardness depth profiles of the coatings (**a**) and dependencies of the average microhardness of remelted zones on the laser operating rate at a constant current 170 A (**b**) and laser current at a constant operating rate 100 mm/min (**c**).

Figure 7a shows microhardness depth profiles of coatings obtained at different processing parameters. The certain fluctuation of hardness values along the depth lines for all the samples can be seen (Figure 7a). For the remelted zones of coatings, it can be associated with the layered microstructure of melted pool cross-section, defined by the morphology of the structural constituents formed and distribution of W-rich carbide precipitates, i.e., carbide phase concentration in different cross-section areas. In the coating zone, which has not been remelted (samples S1, S2, S5, S6), the more expressed swings of hardness values were observed, related to overly large size of Ni-based and WC-based splats compared with an indentation size. Thus, the upper values are attributable to a hardness of WC particles, and the lower—to a Ni-based phase. It should be noted that average pass depth and coating thickness, given above, were measured from the highest convexities of the coating’s surface; therefore, these values slightly differ from those denoted in Figure 7a, which were determined locally. Figure 7b,c show dependencies of average coatings hardness on the laser operating rate at constant current and laser current at a constant rate, respectively. The average hardness values calculated for remelted zones of various samples were in a rather narrow range, from ~1070 HK0.2 and ~1140 HK0.2. Both with a decrease in laser operating speed and with an increase in laser current, a logical slight decrease in hardness is observed, which correlates with the size of the dominant W-rich phase (Figure 7b,c). It can be noted that the lowest hardness values were recorded for S4 samples remelted at the highest power density and heat input.

For samples S4, the penetration depth of which exceeds the coating thickness, a significant iron concentration of ~16 wt.% was recorded, approximately three times higher than that of other samples, and a reduced tungsten concentration, which is associated with a rather high degree of mixing of the coating and substrate materials during remelting. This, along with the dominance of the slightly coarser structure, appears to be the reason for the lower hardness of the remelted layers. For samples S2 and S5, the penetration depth for which is ~2/3 of the thickness of the sprayed coating, as well as for samples S3, the penetration depth for which approximately corresponds to the thickness of the coating, a fairly uniform distribution of the tungsten concentration (characterizing the distribution of the carbide phase) along the coating was established—concentration varies within a narrow range between 19.5 and 20.5 wt.%. It was also found that samples S1 and S6, remelted with the lowest heat input and power density, respectively, are characterized by a more uneven distribution of tungsten concentration in different passes—from ~17 wt.% to ~22 wt.%. This, most likely, can be associated with relatively low melting depth compared with the size of initial WC-based particles; therefore, the effect of certain inhomogeneity of the as-sprayed coating may be more expressed when compared with cases of deeper melting. This, accordingly, entails a more pronounced fluctuation in hardness values. However, it should be noted that the obtained difference in hardness is not significant, and the hardness of all processed coatings is much higher than that of the as-sprayed coating or remelted by using conventional techniques, such as a flame torch, electric furnace, or induction heating (max. ~880 HK, [31]), and it surpasses some results reported for laser cladding and is comparable with the best one (for example, [27]). It can be explained by the more rapid heating and cooling rates typical for pulsed lasers (as compared with continuous one and conventional heating as well), which provided ultrafine microstructure.

### 3.4. Tribology of Laser-Processed Coatings

The hardness testing showed an insignificant difference in obtained hardness. It provides the possibility to set processing parameters based on the required melting depth with no risk to worsen properties. The recommended maximal thickness for flame-sprayed Ni–WC coatings is ~1.5 mm; the normally applied thickness being less. For the tribology testing, four samples’ series were chosen. In terms of obtained melting depth, S3 samples are the most suitable in this work: the depth of the melt pool was slightly over the typical thickness of flame-sprayed Ni–WC coating, providing metallurgical bond and minimal substrate dilution. At S2 and S5 parameters, the melting depth was appropriate as well, providing a rather uniform distribution of W-rich carbide phase and good microhardness; when applied such parameters, the thickness of the as-sprayed layer should be accordingly reduced to provide a metallurgical bond. Samples S2 and S5 were very similar in their microstructure, hardness, and sensitivity to cracks; for testing, one samples’ series S2 was chosen. For the comparison, the S6 samples were tested as well, which have one of the highest average microhardness (~1120 HK0.2), slightly different microstructure morphology due to lower laser power, but showing less uniform W-rich carbides distribution and a too-small melting depth. The as-sprayed coatings R0 were tested as a reference as well.

**Figure 8:**
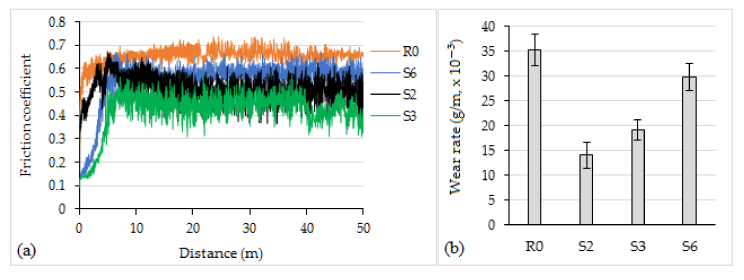
Friction coefficient curves (**a**) and wear rate of coatings (**b**) during a dry sliding test.

It was determined that the friction coefficient of the as-sprayed coatings was ~0.66 and it was improved after the laser processing. For tested laser-processed samples, the following values of friction coefficient were established: for S2—~0.52; S3—~0.44; S6—~0.58 (Figure 8a). Such improvement at higher coatings hardness can be associated with the formation of finer, monolithic, and more uniform structure after laser processing, as compared with as-sprayed state. The transformation of carbide from type WC/W_2_C (in the as-sprayed layer, as was established earlier) into (W, Cr, Ni, Fe)C (in laser-remelted coatings) could have some effect as well. The coatings resistance to wear was improved as well: the average wear rate of tested laser-processed surfaces was ~15.0 × 10^−3^, ~19.1 × 10^−3^, and 29.9 × 10^−3^ g/m, and it was ~2.4 times, ~80%, and ~18% less than that of the as-sprayed coatings, respectively (Figure 8b). S2 and S3 showed better results, while S6 coatings, despite having rather high hardness, showed the worst wear resistance among the tested laser-processed samples. Larger mass loss during testing and higher friction coefficient of the S6 series can be related with a too-small melting depth and width, resulting in formation of noncompletely remelted surface at the applied pass overlapping. As a result, small pieces of the coating top layer crumbled under the applied load during tribology testing, increasing mass loss and worsening sliding conditions.

## 4. Conclusions

In the present study, the flame-sprayed NiCrCoFeCSiB/WC coatings were postprocessed with pulsed Nd:YAG laser, and the effect of the processing parameters on the coatings’ cracking, pass geometry, microstructure, hardness, and tribology were analyzed. The following conclusions were drawn from the results of experimental research presented in this paper:

1. The laser peak power density between 4.00 × 10^6^ and 5.71 × 10^6^ W/cm^2^ and laser operating speed between 100 and 400 mm/min provided processing of flame-sprayed NiCrCoFeCSiB/WC coatings in a melting mode. The width of individual pass changed insignificantly with varying laser processing parameters and ranged between 1050 and 1150 μm (exception—the regime at the lowest power density and heat input); the depth of pass was in strong near-linear dependence on both the power and speed and ranged between ~290 and ~1330 μm.
2. The laser-processed coating most sensitive to cracking was obtained at the highest power density, 5.71 × 10^6^ W/cm^2^, and the lowest operating speed, 100 mm/min. A significant reduction in cracks appearance was observed with reducing power density.
3. The pulsed laser processing provides a monolithic remelted coating layer with a microstructure of W-rich dendrites in Ni-based matrix, where size and distribution of W-rich dendrites periodically vary across remelted layer depth. The composition of W-rich dendrites of different sizes and shapes does not differ significantly and can be attributed to a carbide of type (W, Cr, Ni, Fe)C.
4. The prevailing of difference in size microstructure in coatings laser-processed at different parameters led to a slight variation in microhardness from ~1070 to ~1140 HK0.2, that is ~20–30% higher compared with coatings remelted using conventional techniques, such as flame, furnace, or induction heating, and is comparable with the best results reported for laser cladding (1200 HV0.2). The trend of a slight coatings hardness increase at higher laser processing speed and lower laser power density was established.
5. The friction coefficient and wear rate of coatings during dry sliding were reduced by up to ~30% and up to ~2.4 times, respectively, after laser processing.
6. Very small melting depth (~300 μm) resulted in a less-uniform carbide phase distribution, formation of noncompletely remelted coating surface, and increased friction coefficient and wear rate during tribology test (as compared with other laser-processed coatings). Too-high laser density and heat input resulted in too-deep melting, higher sensitivity to cracking, and too-high level of coating and substrate mixing. Therefore, it is reasonable to balance power with appropriate operating speed to provide pass depth close to the thickness of the deposited layer. Based on the obtained dependencies for melted pool geometry, the optimal regimes may be determined more precisely, providing minimal substrate dilution and metallurgical bond. In addition, the step between adjacent passes may be reduced, providing larger pass overlapping. It is reasonable, as well, to continue study aiming to evaluate the effect of pre- and postheating procedures on coatings’ sensitivity to cracking and possible hardness reduction.

## Figures and Tables

**Table 1:** Coding of laser-processed samples.

Sample Code	Laser Current, A	Peak Power Density, W/cm^2^	Laser Operating Speed, mm/min
S1	170	5.71 × 10^6^	400
S2	170	5.71 × 10^6^	300
S3	170	5.71 × 10^6^	200
S4	170	5.71 × 10^6^	100
S5	145	4.85 × 10^6^	100
S6	120	4.00 × 10^6^	100

**Table 2:** Chemical composition of W-rich phases and Ni-based matrix in points showed in Figure 6 (by EDS).

Element	W-Rich Phase (in at.%)						Ni-Based Matrix (in wt.%)					
	Sp.1	Sp.2	Sp.3	Sp.4	Sp.5	Sp.6	Sp.7	Sp.8	Sp.9	Sp.10	Sp.11	Sp.12
B	-	-	-	-	-	-	+	+	+	+	+	+
C	48.01	53.17	51.71	48.10	53.49	50.85	+	+	+	+	+	+
O	-	-	-	-	-	-	+	+	+	+	+	+
Si	1.43	-	1.37	1.61	-	-	0.46	0.66	1.48	1.04	0.68	1.23
Cr	18.85	17.40	16.97	16.96	16.54	17.49	16.06	15.91	17.60	17.09	16.25	16.31
Fe	3.34	4.41	5.24	5.87	4.04	4.21	15.18	15.39	10.99	11.37	12.59	12.35
Co	-	-	-	-	-	-	6.44	6.03	2.83	3.89	5.19	4.99
Ni	17.96	13.74	15.17	16.76	16.67	17.65	60.99	61.24	65.80	65.37	64.57	64.49
W	10.41	11.40	9.53	10.70	9.26	9.80	0.87	0.76	1.30	1.25	0.73	0.62
Total	100.00	100.00	100.00	100.00	100.00	100.00	100.00	100.00	100.00	100.00	100.00	100.00

+ EDS analysis showed the presence of C and B in all the samples. Since the sensitivity of EDS to light elements is low, B and C were eliminated from composition to avoid results warping.
